# Susceptibility of Raccoon Dogs for Experimental SARS-CoV-2 Infection

**DOI:** 10.3201/eid2612.203733

**Published:** 2020-12

**Authors:** Conrad M. Freuling, Angele Breithaupt, Thomas Müller, Julia Sehl, Anne Balkema-Buschmann, Melanie Rissmann, Antonia Klein, Claudia Wylezich, Dirk Höper, Kerstin Wernike, Andrea Aebischer, Donata Hoffmann, Virginia Friedrichs, Anca Dorhoi, Martin H. Groschup, Martin Beer, Thomas C. Mettenleiter

**Affiliations:** Friedrich-Loeffler-Institut, Greifswald-Insel Riems, Germany

**Keywords:** raccoon dogs, COVID-19, SARS-CoV-2, susceptibility, transmission, respiratory infections, severe acute respiratory syndrome coronavirus 2, 2019 novel coronavirus disease, coronavirus disease, zoonoses, viruses, coronavirus, Nyctereutes procyonoides

## Abstract

Raccoon dogs might have been intermediate hosts for severe acute respiratory syndrome–associated coronavirus in 2002–2004. We demonstrated susceptibility of raccoon dogs to severe acute respiratory syndrome coronavirus 2 infection and transmission to in-contact animals. Infected animals had no signs of illness. Virus replication and tissue lesions occurred in the nasal conchae.

Severe acute respiratory syndrome coronavirus 2 (SARS-CoV-2) emerged in Wuhan, China, at the end of 2019. Researchers have identified close relatives to SARS-CoV-2 in bats (*1*) and pangolins (order Pholidota) (*2*,*3*). Whether the pandemic was initiated by direct transmission from bats or through an intermediate mammalian host is still under debate (*4*). During the 2002–2004 severe acute respiratory syndrome pandemic, researchers documented the causative virus in raccoon dogs (*Nyctereutes procyonoides*) in China, indicating that these animals might have been intermediate hosts for the virus (*5*). Fur producers in China own >14 million captive raccoon dogs, accounting for »99% of the global share of raccoon dogs (6) (Appendix Figure 1). However, whether these animals are susceptible to SARS-CoV-2 is unknown. Using our established study design (*7*), we characterized susceptibility, viral shedding, transmission potential, serologic reactions, and pathologic lesions of raccoon dogs after experimental SARS-CoV-2 infection.

## The Study

**Figure 1:**
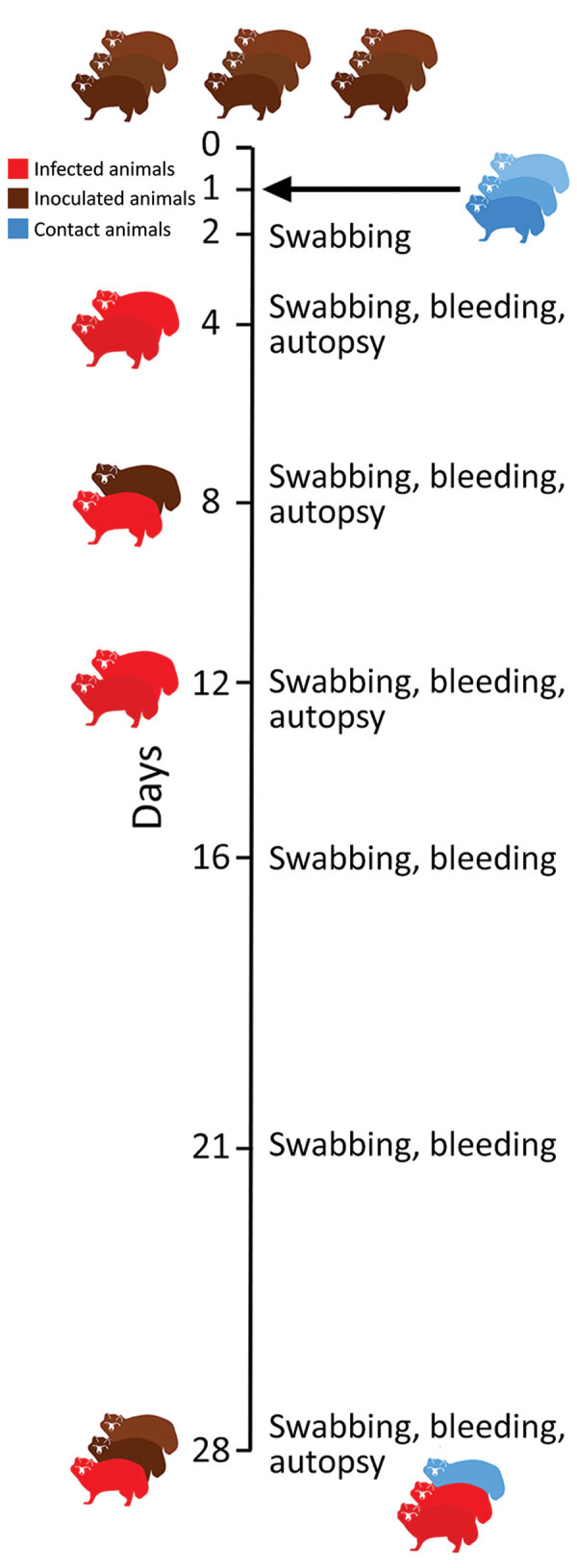
Study design for experimental infection of raccoon dogs with severe acute respiratory syndrome coronavirus 2. Outline of the in vivo experiment with an observation period of 28 days; 9 animals were inoculated intranasally with 10^5^ 50% tissue culture infectious dose/mL, and 3 naive direct contact animals were introduced 24 hours later. On days 4, 8 and 12, two raccoon dogs were euthanized and autopsied. All remaining animals were euthanized on day 28. Red indicates infected animals.

We intranasally inoculated 9 naive raccoon dogs with 10^5^ 50% tissue culture infectious dose (TCID_50_) SARS-CoV-2 2019_nCoV Muc-IMB-1. We introduced 3 naive animals 24 hours after inoculation to test for direct transmission (Figure 1). We sorted animals into 4 groups of 3 individual cages separated by meshed wire and placed each naive contact animal between 2 inoculated animals (Appendix Figure 2). We also used 2 naive animals as controls. Although several animals (animal nos. 4, 5, and 10) were slightly lethargic 4 days after inoculation, none of the exposed or contact animals had fever, weight loss, or other signs of clinical infection.

**Figure 2:**
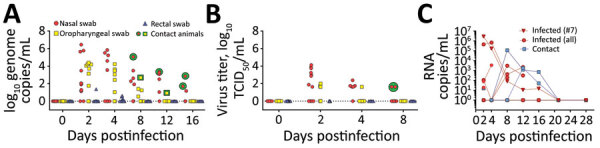
Detection of severe acute respiratory syndrome coronavirus 2 in swab samples from experimentally infected raccoon dogs. A) Viral genome loads in swab samples isolated on Vero E6 cells; B) viral genome loads in virus titers isolated on Vero E6 cells. Two replicates per sample were analyzed. C) Individual viral loads of nasal swab specimens taken from infected and contact animals.

To monitor viral shedding, we collected nasal, oropharyngeal, and rectal swab samples on days 2, 4, 8, 12, 16, 21, and 28. We measured viral RNA by quantitative reverse transcription PCR and the levels of infectious virus by titration on Vero E6 cells (Figure 2). We observed viral shedding in 6 (66.7%) of 9 inoculated animals. Because we did not detect viral shedding in animal nos. 4, 8, and 9 during the 28-day observation period, we concluded that these animals were not successfully infected. The infected animals shed virus in nasal and oropharyngeal swab samples on days 2–4; we found viral RNA in nasal swab samples until day 16 (animal no. 7). The mean viral genome load was 3.2 (range 1.0–6.45) log_10_ genome copies/mL for nasal swab samples, 2.9 (range 0.54–4.39) log_10_/mL for oropharyngeal swab samples, and 0.71 (range 0.31–1.38) log_10_/mL for rectal swab samples. Titrations showed the same trend; viral titers peaked at 4.125 log_10_ TCID_50_/mL in nasal swabs on day 2. We successfully isolated virus from all except 2 RNA-positive samples that had a cycle threshold of <27. However, we could not isolate virus from samples that had a cycle threshold >27 (Appendix Figure 3).

We detected infection in 2 (66.7%) of 3 contact animals (nos. 10 and 11) (Figure 2; Appendix Figure 2). We first detected viral RNA in animal no. 10 on day 8 (i.e., 7 days after contact). Viral shedding, mainly in nasal secretions, lasted until day 16 (15 days after contact), and we identified viral titers of 1.625 log_10_ TCID_50_/mL in nasal swab samples on day 8 (7 days after contact). One contact raccoon dog (no. 12) remained negative for SARS-CoV-2 because infection did not develop in either of his inoculated cage neighbors (nos. 8 and 9) (Appendix Figure 2).

On days 4, 8, 12, and 28, we euthanized and conducted autopsies on 2 animals in sequential order. We tested tissues and body fluids for SARS-CoV-2 RNA and replicating virus (Appendix Figure 4). We found viral loads of up to 4.87 log_10_ genome copies/mL in the nasal mucosa on day 4 but only minute amounts in other organs. We cultivated infectious virus from the nasal conchae of animal nos. 1 (2.86 log_10_ TCID_50_/mL) and 2 (1.63 log_10_ TCID_50_/mL). None of the lung samples tested positive for viral RNA.

In the autopsies, we did not find gross lesions definitively caused by SARS-CoV-2 infection. We used hematoxylin and eosin staining on tissues taken at autopsy on days 4, 8, and 12 to identify mild rhinitis affecting the respiratory and olfactory regions in all infected animals (Appendix Figure 5) but not in negative controls. We used immunohistochemical tests to verify the presence of intralesional SARS-CoV-2 antigen in the nasal respiratory and olfactory epithelium on days 4 and 8 (Appendix Figure 5). We did not find the antigen at later time points, possibly because of virus clearance or the limited sensitivity of the immunohistochemical test. We did not detect histopathologic lesions nor viral antigen in animal no. 4, which had not been successfully infected, on day 8. On day 28, 1 infected (no. 7) and 1 contact animal (no. 10) had histologic lesions indicative of SARS-CoV-2 replication in the nasal conchae (Appendix Figure 6). We still detected viral RNA but no antigen. We did not detect further lesions definitively caused by SARS-CoV-2-infection. All other tissues tested negative for SARS-CoV-2 antigen (Appendix).

We took serum samples on days 4, 8, 12, 16, 21, and 28. We tested these samples for antibodies against SARS-CoV-2 using the indirect immunofluorescence assay and virus neutralization test as described (*7*). We detected SARS-CoV-2–specific antibodies in 4 (57.1%) of 7 inoculated animals on day 8 using ELISA (Appendix Figure 7, panel A) and indirect immunofluorescence assay (>1:64) (Table). Titers increased to 1:1,024 on day 28 (animal no. 7). We observed neutralizing antibodies in 2 of the infected animals (nos. 6 and 7) as early as day 8 (animal no. 6, 1:5.04) (Table). The highest titer of neutralizing antibodies was 1:12.7 (found in no. 6 on day 12, and no. 7 on day 28). We characterized SARS-CoV-2–specific immunoglobulins, revealing that IgM, IgG, and IgA developed within 8 days after infection; IgM levels peaked on day 8 and IgG on day 12 (Appendix Figure 7, panels B–G). On days 8 and 12, we also detected antibodies specific for the receptor-binding domain of SARS-CoV-2 in saliva samples from animals that developed serum antibodies (Appendix Figure 7, panels H–I). In contrast to SARS-CoV-2 isolates from infected ferrets (*7*), the isolates from nasal swabs of infected raccoon dogs (animal no. 2 on day 2 and no. 10 on day 8) demonstrated 100% sequence identity to the inoculum.

**Table:** Serologic response of raccoon dogs to experimental SARS-CoV-2 infection, by day after inoculation*

Animal no.	Day 8			Day 12			Day 16			Day 21			Day 28	
	iIFA	VNT		iIFA	VNT		iIFA	VNT		iIFA	VNT		iIFA	VNT
Inoculated														
1														
2														
3	1:128	<1:4												
4	<1:20	<1:2												
5	1:64	<1:2		1:64	<1:2									
6	1:128	1:5.04		1:64	1:12.7									
7	1:128	<1:4		1:64	<1:2		1:64	1:4		1:128	1:10.08		1:1,024	1:12.7
8	<1:20	<1:2		<1:20	<1:2		<1:20	<1:2		<1:20	<1:2		<1:20	<1:2
9	<1:20	<1:2		<1:20	<1:2		<1:20	<1:2		<1:20	<1:2		<1:20	<1:2
In-contact														
10	<1:20	<1:2		<1:20	<1:2		1:64	<1:2		1:128	<1:4		1:512	<1:4
11	<1:20	<1:2		<1:20	<1:2		1:64	<1:2		1:128	1:5.04		1:256	<1:4
12	<1:20	<1:2		<1:20	<1:2		<1:20	<1:2		<1:20	<1:2		<1:20	<1:2

*No serologic response recorded for days 0 and 4. Animal nos. 4, 8, and 9 did not show signs of infection. Each day, 2 raccoon dogs were euthanized and autopsied. iIFA, indirect immunofluorescence assay; SARS-CoV-2, severe acute respiratory syndrome coronavirus 2; VNT, virus neutralization test.

## Conclusions

Our experimental study demonstrates that raccoon dogs are susceptible to SARS-CoV-2 infection and can transmit the virus to direct in-contact animals. In our study, raccoon dogs had only subtle clinical signs. We found evidence of viral replication and tissue lesions in only the nasal conchae.

Increasing evidence supports the potential of carnivore species, including farmed fur animals, to become infected by SARS-CoV-2 (*8*–*12*). This transmission could eventually cause zoonotic infections in humans (B.B. Oude Munnink, unpub. data, https://www.biorxiv.org/content/10.1101/2020.09.01.277152v1). Our results indicate that affected farms might be reservoirs for SARS-CoV-2. Thus, efficient and continuous surveillance should target susceptible animals, including raccoon dogs, especially in China, which is a key player in global fur production (*6**)*. We also need to initiate large-scale epidemiologic field studies with historic samples that might elucidate the role of farmed animals in the current pandemic.

This article was preprinted at https://www.biorxiv.org/content/10.1101/2020.08.19.256800v1.

Appendix Further information on methods and results of experimental SARS-CoV-2 infection in raccoon dogs.
