# Supplementary material for: Susceptibility of Raccoon Dogs for Experimental SARS-CoV-2 Infection
Source: Emerg Infect Dis. 2020 Dec;26(12):2982–5. doi: 10.3201/eid2612.203733 (PMC7706974; doi:10.3201/eid2612.203733)
Supplement: Appendix — Further information on methods and results of experimental SARS-CoV-2 infection in raccoon dogs. [file 20-3733-Techapp-s1.pdf]

Article DOI: https://doi.org/10.3201/eid2612.203733

Susceptibility of Raccoon Dogs for Experimental SARS-CoV-2 Infection

Appendix

Material and Methods

Animals and Study Design

The experimental design followed the general setup of a previous study (*1*). Fourteen adult, male (n = 4) and female (n = 10) raccoon dogs originating from a commercial farm (Ferma Hodowlana Lisow, 64–050 Wielichowo, Poland) were used. Animals were transported according to European regulations by an international company specialized for transportation of zoo animals (MSO International Zoo Services GmbH; TRACES-No: DE 071. 380.450.267).

All animals tested negative by quantitative reverse transcription PCR (RT-qPCR) and antibody tests (ELISA, indirect immunofluorescence assay [iIFAT], virus neutralization test [VNT]) for SARS-CoV-2 before the inoculation.

All raccoon dogs had been vaccinated against distemper, adenovirus and parvovirus (Eurican® SHP, Merial, France). Animals were kept in individual stainless-steel cages (1.5 m × 0.95 m × 2.0 m) in 4 separate segments at 20°C room temperature, 60%–80% humidity and a 12 hr/12 hr (35% dimming during night modus) lighting control within a fan forced draft ventilation equipped Biosafety Level 3 animal facility at the Friedrich-Loeffler-Institut (FLI). Water was offered ad libitum. Animals were fed daily with 400 g commercially produced feed for farmed foxes and raccoon dogs (Schirmer und Partner GmbH Co KG, Germany; Michael Hassel GmbH, Langenargen, Germany). The diet was supplemented with vitamins, minerals, and items like 1-day-old chickens (*2*). The general health status of all animals, feed uptake and defecation were recorded daily. The body weight and temperature of all animals were measured before inoculation and at days 2, 4, 8, 12, 16, 21, and 28.

Nine raccoon dogs (3 males, 6 females) were infected intranasally with 10^5 50% tissue culture infectious dose SARS-CoV-2 2019_nCoV Muc-IMB-1. The inoculum of 2 × 1 mL was administered to both nostrils using a pipette. To test viral transmission by direct contact, 3 naïve sentinel animals (all female) were added 24 hours post inoculation.

Animals were anesthetized for challenge, blood collection, and swabbing with 1.0 mL of Zoletil (combination of Tiletamin and Zolazepam, Virbac, France). Nasal, oropharyngeal and rectal swabs were taken at 2, 4, 8, 12, 16, 21 and 28 days post infection (dpi), blood was taken from the large superficial veins of the lower (Vena saphena) at 4, 8, 12, 16, 21 and 28 dpi. Two animals each were euthanized and autopsied at day 0 (control #1, #2) day 4 (animals #1, #2), day 8 (animals #3, #4) and day 12 pi (animals #5, #6). The remaining inoculated animals (animals #7–9) and the contacts (animals #10–12) were euthanized 28 dpi. For euthanasia, animals were first anaesthetized with Zoletil followed by cardiac bleeding.

All animals were subjected to autopsy for macroscopic evaluation and tissue sampling. The animal experiments were evaluated and approved by the ethics committee of the State Office of Agriculture, Food Safety, and Fishery in Mecklenburg–Western Pomerania (LALLF M-V: LVL MV/TSD/7221.3–2-010/18–12).

Virus and Cells

SARS-CoV-2 isolate 2019_nCoV Muc-IMB-1 was kindly provided by R. Woelfel (German Armed Forces Institute of Microbiology, Munich, Germany). The complete sequence of this isolate is available through GISAID under the accession ID_EPI_ISL_406862 and designation "hCoV-19/Germany/BavPat1/2020." The virus was propagated once in Vero E6 cells in a mixture of equal volumes of Eagle MEM (Hanks' balanced salts solution) and Eagle MEM (Earle's balanced salts solution) supplemented with 2 mM L-Glutamine, nonessential amino acids, adjusted to 850 mg/L, NaHCO3, 120 mg/L sodium pyruvate, 10% fetal bovine serum (FBS), pH 7.2. No contaminants were detected within the virus stock preparation by metagenomic analysis employing previously published high throughput sequencing procedures Ion Torrent S5XL instrument (C. Wylezich, unpub. data, https://www.biorxiv.org/content/10.1101/2020.06.30.181446v1) (*3*) and the sequence identity of the passaged virus (study accession number: PRJEB39640) was confirmed. The virus was harvested after 72 h, titrated on Vero E6 cells and stored at −80°C until further use. All work was performed in a Class II Biosafety Cabinet under Biosafety Level 3 conditions at the Friedrich-Loeffler-Institut.

Virus Detection

Virus and viral RNA were detected as described before (*1*). Tissues were homogenized in 1 mL cell culture medium (see above) and a 5 mm steel bead in a TissueLyser (Qiagen, Hilden, Germany). Fecal samples were vortexed in sterile sodium chloride (0.9%) and the supernatant was sterile filtered (22 μm) after centrifugation. Swab samples were transferred into 1 mL of serum-free tissue culture media and further processed

after 30 min shaking. From 100 µL of this material, total RNA were extracted using the NucleoMagVet kit (Macherey&Nagel, Düren, Germany) according to manufacturer's instructions, in an elution volume of 100 µL.

SARS-CoV-2 RNA was detected by an E-gene based RT-qPCR (*4*). The RT-qPCR reaction was prepared using the AgPath-ID-One-Step RT-PCR kit (Thermo Fisher Scientific, Waltham, Massachusetts, USA) in a volume of 12.5 µL including 1 µL of β-Actin-mix2-HEX as internal control and 2.5 µL of extracted RNA. The reaction was performed for 10 min at 45°C for reverse transcription, 5 min at 95°C for activation, and 42 cycles of 15 sec at 95°C for denaturation, 20 sec at 57°C for annealing, and 30 sec at 72°C for elongation. Fluorescence was measured during the annealing phase. All RT-qPCRs were performed on a BioRad real-time CFX96 detection system (Bio-Rad, Hercules, CA, USA). Absolute quantification was calculated per RT-PCR reaction using a dilution series of a standard which was quantified by the QX200 Droplet Digital PCR System in combination with the 1-Step RT-ddPCR Advanced Kit for Probes (BioRad, Hercules, CA, USA).

RT-qPCR positive swabs and tissue samples were subjected to virus isolation and titration on Vero E6 cells. Briefly, the cells were inoculated in duplicate and the endpoint titer was calculated as $TCID_{50}$ per milliliter applying the Spearman-Kärber algorithm.

Nasal swab samples from raccoon dog #2 (2 dpi) and from contact animal #10 (8 dpi) were subjected to high-throughput sequencing and compared to the inoculum (study accession number: PRJEB39640) by employing previously published high throughput sequencing procedures using Ion Torrent S5XL instrument (C. Wylezich, unpub. data, https://www.biorxiv.org/content/10.1101/2020.06.30.181446v1) (*3*).

Detection of SARS-CoV-2 Reactive Antibodies

For iIFAT, confluent Vero E6 cells in a 96 well plate were infected with 0.1 multiplicity of infection (MOI) of SARS-CoV-2 or overlaid with cell culture medium for negative control cells. After 24 h, cells were fixed with 4% paraformaldehyde and permeabilized with 0.5% Triton-X-100. Serum samples were heat inactivated at 56°C for 30 min. For antibody detection, 50 µL of a 2-fold dilution series of the serum samples (starting from 1:20) were added in parallel to the SARS-CoV-2 positive and negative cells. After 1 h incubation, cells were washed and incubated for 1h with a goat-α-dog-IgG-Fluorescein isothiocyanate antibody (1:250, Bethyl, Texas, USA). After final washing, cells were analyzed by fluorescence microscopy.

For VNT, 50 µL of medium containing $10^{3.3}$ $TCID_{50}$ SARS-CoV-2 were mixed with 50 µL of serially diluted serum. Each sample was tested in triplicates. After 1 h incubation at 37°C the mixture was transferred to confluent Vero E6 cells in a 96 well plate. Viral replication was assessed after 5 days at 37°C, 5% CO_2 by the detection of cytopathogenic effects (CPE).

For ELISA, the SARS-CoV2 RBD-SD1 domain (amino acids 319–519 of the SARS-CoV2 Spike ectodomain) was amplified from a codon-optimized synthetic gene (GeneArt, Thermo Scientific). The construct was cloned into the expression vector pEXPR103 (iba lifesciences) in frame with an N terminal modified mouse Ig kappa light chain signal peptide and a C-terminal double Strep tag. Expi293 cells were grown in Expi293 expression medium (Thermo Scientific) and polycarbonate Erlenmeyer flasks (Corning) at 37°C, 8% CO_2, 125 rpm. For transfection, cell density was adjusted to 2×10^6 cells/mL and a total volume of 70 mL was transfected with 70 µg of plasmid DNA using the ExpiFectamine293 transfection kit (Thermo Scientific) according to the manufacturer´s instructions. The cells were subsequently incubated at 37°C, 8% CO_2, 125 rpm. The supernatant was harvested 5 days after transfection by centrifugation at 6000 × g for 20 min at 4°C. Biotin was blocked by addition of BioLock (iba lifesciences) as recommended, and the supernatant was purified using Strep-Tactin XT Superflow high capacity resin (iba lifesciences) according to the protocol of the manufacturer. The proteins were eluted with 50 mM Biotin (in 100 mM Tris-HCl, 150 mM NaCl, 1mM EDTA; pH 8.0) and stored at −80°C until further use. Medium binding ELISA plates (Greiner Bio-One GmbH, Germany) were coated with 100 ng/well of the SARS-CoV-2 RBD overnight at 4°C in 0.1 M carbonate buffer at pH 9.6 or treated with the coating buffer only. Thereafter, the plates were washed three times using Tris-buffered saline with Tween (TBST) and blocked for 1 h at 37°C using 5% skimmed milk in phosphate-buffered saline (PBS). The serum samples were diluted 1:100 in TBST and incubated on the coated and uncoated wells for 1 h at room temperature followed by three washes using TBST. The saliva samples were used undiluted. Reactivity was shown by adding a multi species conjugate (SBVMILK; IDvet, France) diluted 1:80 (serum) or 1:10 (saliva). After an incubation period of 1 h at room temperature, the plates were washed again and Tetramethylbenzidine (TMB) substrate (IDEXX, Switzerland) was added. The ELISA readings were taken at a wavelength of 450 nm on a Tecan Spectra Mini instrument (Tecan Group Ltd, Switzerland). The measurements were normalized to the respective samples tested on wells treated only with the coating buffer.

SARS-CoV-2 specific immunoglobulins (Ig) were comparatively investigated in serum samples and saliva of raccoon dogs by ELISA using exactly the same SARS-CoV-2

RBD-SD1 antigen-coated plates, serum dilutions, washing and dilution buffers, TMB substrate, incubation periods and ELISA-Reader as described above. After the incubation of the serum samples or saliva and the following washing, dog-specific, horseradish-peroxidase (HRP) labeled Ig antibodies (goat-α-dog-IgA 1:1,000 for saliva and 1:5,000 for serum; goat-α-dog-IgM1 1:15,000; goat-α-dog-IgG, goat-α-dog-IgG1, goat-α-dog-IgG2 all 1:20,000; Bethyl Laboratories INC) were added and incubated for 1 h at RT. Antibodies were diluted in TBST.

Pathology: Autopsy, Histopathology, Immunohistochemistry

Full autopsy was performed on all animals under BSL3 conditions. The following tissues were collected and fixed in 10% neutral-buffered formalin and trimmed for paraffin embedding: nasal atrium (non-respiratory region), nasal conchae (respiratory and olfactory region, cross sections approximately every 5 mm after decalcification), soft palate, tonsil, parotid and mandibular salivary gland, trachea (upper and lower third), lung (inflated with formalin, cross sections of the right cranial, medial, caudal and accessory lobe), tracheobronchial lymph node, heart, liver with gall bladder, spleen, stomach (cardiac, fundic, pyloric region), intestine (duodenum, jejunum, ileum, colon, cecum, rectum), pancreas, kidney, ureter, urinary bladder, adrenal gland, skeletal muscle, skin, and brain (3 coronal section with lobus olfactorius, hippocampus and cortex, cerebellum und medulla oblongata). In addition, blood (5 mL EDTA, rest serum), urine (puncture at autopsy), cerebrospinal fluid, and bronchio-alveolar lavage (left lobe, using 10 mL PBS), were taken for viral RNA detection and virus isolation.

Tissues were cut at 3 μm sections and stained with hematoxylin and eosin for light microscopical examination. Evaluation and interpretation was performed by a board-certified pathologist (DiplECVP). The severity of lesions and the distribution of SARS-CoV-2 antigen was recorded on an ordinal scoring scale with scores 0 = no lesion/antigen, 1 = rare, affected cells/tissue <5% per slide; 2 = multifocal, 6%–40% affected; 3 = coalescing, 41%–80% affected; 4 = diffuse, >80% affected.

The nasal mucosa was particularly scored for cellular degeneration and necrosis, loss of epithelium, intraluminal debris, mucosal infiltration and transmigration, endothelial activation (swelling), edema, hemorrhage, reepithelization/squamous metaplasia and granulation tissue formation. All lung sections were scored for lesion on the pleural surface, alveolar collapse, endothelial activation (swelling), edema (interstitial, bronchial, peribronchial, perivascular, alveolar), congestion, inflammatory infiltrates (interstitial,

peribronchial, perivascular), increased numbers of alveolar macrophages, necrosis (bronchial, alveolar), squamous metaplasia, hyperplasia (bronchial, pneumocyte type 2), presence of multinucleated giant cells, cellular debris in bronchial lumina, alveolar emphysema.

Statistical Information

No statistical methods were used. The experiments were not randomized and the investigators were not blinded to allocation during experiments and outcome assessment. All samples for downstream virological and serologic analyses were blinded. All data were analyzed and visualized using GraphPad Prism Version 7.0 (GraphPad Software, San Diego, CA, USA).

Appendix Pathology Data

Histopathology identified mild rhinitis at 4, 8, and 12 dpi in all successfully infected animals (#1–3, #5, #6). The olfactory, caudal region of the nasal cavity was more consistently affected compared to the respiratory, cranial region and included degeneration, necrosis and loss of the respiratory and olfactory epithelium, presence of intraluminal cellular debris, degeneration and necrosis of the submucosal glands, mucosal edema, endothelial swelling and acute, submucosal hemorrhage (Appendix Figure 5, panels A–B). At 4 dpi, mainly neutrophils with fewer macrophages and lymphocytes were found, later lesions showed predominantly lymphocytes and fewer neutrophils and macrophages. Mucosal coagulative necrosis with early reepithelization and granulation tissue formation was present in one case (Appendix Figure 5, panel C, 8 dpi). At 28 dpi, one infected (#7) and one contact animal (#10) showed lesions indicative for previous viral replication sites in the nasal conchae. Viral RNA was still present, but no viral antigen was found (Appendix Figure 6). The remaining animals at 28 dpi did not exhibit lesions in the nasal cavity, potentially because (I) full resolution of previous lesions occurred, (II) focal lesions were not present in the slides evaluated, (III) animals were not infected, or (IV) animals were infected but did not develop lesions.

Immunohistochemistry verified the presence of viral antigen only in the nasal conchae. Lesion associated antigen was found to be oligofocal at day 4 (animal #1, #2) in the respiratory and olfactory epithelium and to a lesser extent at day 8 (animal #3) only in the olfactory epithelium (Appendix Figure 5, panels D–F). No viral antigen could be found at 12 dpi and 28 dpi, potentially due to previous virus clearance or the detection limit of

immunohistochemistry. Neither histopathologic lesions nor viral antigen were detected in animal #4 (8 dpi) in the nasal cavity.

No further lesions were detected in the upper and lower respiratory tract, the gastrointestinal tract, the urinary tract, brain, and main parenchyma that could be assigned to the SARS-CoV-2-infection. Except for the nasal conchae, all other tissues tested negative for SARS-CoV-2 antigen.

Appendix Pathology Data (Findings interpreted as Non-Related to SARS-CoV-2 infection)

At autopsy, several animals exhibited oligofocal pulmonary atelectasis. Histopathology identified alveolar collapse without significant inflammatory infiltrates in affected regions, but also in otherwise macroscopically inconspicuous lungs. The histologic alteration was also found in negative control animals as well as in animals inoculated with SARS-CoV-2 that were tested negative for the virus and seroconversion throughout the experiment. Viral antigen could not be detected at any time point in any lung tissue tested. Thus, this finding was interpreted as not-related to SARS-CoV-2 infection.

Animal #6 showed rare necrosis of bronchial and alveolar epithelium, cellular debris in bronchial lumina, multifocal to coalescing hyperplasia, squamous metaplasia of the bronchial epithelium, and multifocal to diffuse peribronchial edema. Inflammation in the lung but also in the gall bladder was characterized by diffuse, mainly lymphocytic and eosinophilic inflammatory infiltrates. The right, medial lobe of the liver of this animal was affected by cholangiocellular carcinoma. Taken together, the findings in the lung, gallbladder and liver are interpreted to be related to a chronic parasitic burden, most likely trematodes.

Additional findings in other tissues tested did not exceed lesions found in negative control animals or were interpreted as background findings and included: pulmonary congestion, peribronchial aggregates of macrophages containing dark brown–black pigment (anthracosis), focal crypt abscesses in the large intestine, serocellular crusts on and lymphocytic transmigration of the tonsillar mucosa, aggregation of lymphocytes in the apical propria of the small and large intestine, glandular cystic dilation in the gastric mucosa, hepatocellular degeneration or single cell necrosis with minimal inflammatory reaction, hemosiderosis in the spleen.

References

1. Schlottau K, Rissmann M, Graaf A, Schön J, Sehl J, Wylezich C, et al. SARS-CoV-2 in fruit bats, ferrets, pigs, and chickens: an experimental transmission study. Lancet Microbe. 2020;1:e218–25. PubMed https://doi.org/10.1016/S2666-5247(20)30089-6

2. Freuling CM, Eggerbauer E, Finke S, Kaiser C, Kaiser C, Kretzschmar A, et al. Efficacy of the oral rabies virus vaccine strain SPBN GASGAS in foxes and raccoon dogs. Vaccine. 2019;37:4750–7. PubMed https://doi.org/10.1016/j.vaccine.2017.09.093

3. Wylezich C, Papa A, Beer M, Höper D. A versatile sample processing workflow for metagenomic pathogen detection. Sci Rep. 2018;8:13108. PubMed https://doi.org/10.1038/s41598-018-31496-1

4. Etievant S, Bal A, Escuret V, Brengel-Pesce K, Bouscambert M, Cheynet V, et al. Performance assessment of SARS-CoV-2 PCR assays developed by WHO referral laboratories. J Clin Med. 2020;9:1871. PubMedhttps://doi.org/10.3390/jcm9061871

5. ACTAsia.org. China's fur trade and its position in the global fur industry. 2019 Jul [cited 2019 Jul 7]. https://www.actasia.org/wp-content/uploads/2019/10/China-Fur-Report-7.4-DIGITAL-2.pdf</eref>

6. International Union for Conservation of Nature and Natural Resources. Raccoon dog. 2016 [cited 2019 Jul 7] https://www.iucnredlist.org/species/14925/85658776

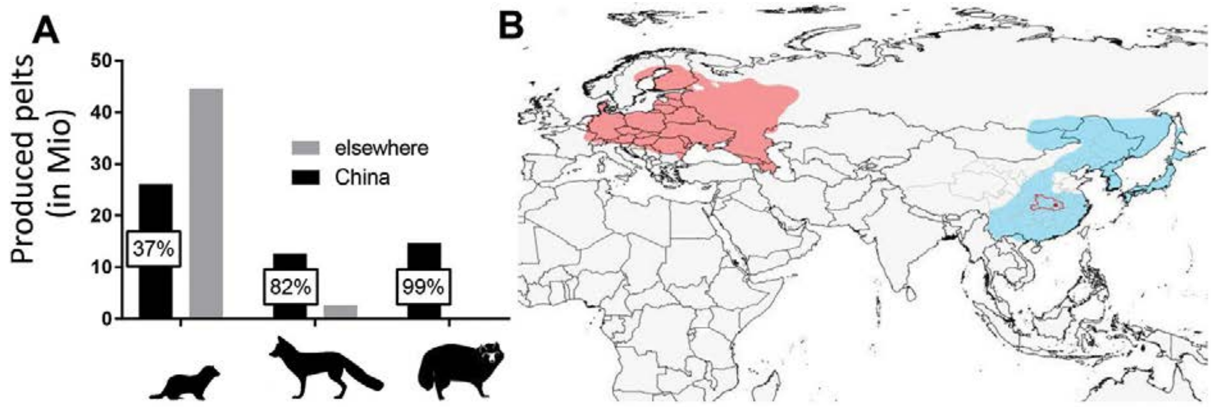

Appendix Figure 1. Relevance of fur production, worldwide distribution of raccoon dogs, and phylogenetic overview of potential animal hosts for SARS-CoV-2. (A) Total number of produced pelts of minks, foxes and raccoon dogs in China and elsewhere in the world. Black indicates China's proportion of the global share (*5*). (B) Distribution of the raccoon dog. Blue indicates traditional distribution; orange indicates anthropogenic expansion; red indicates Hubai, China (*6*).

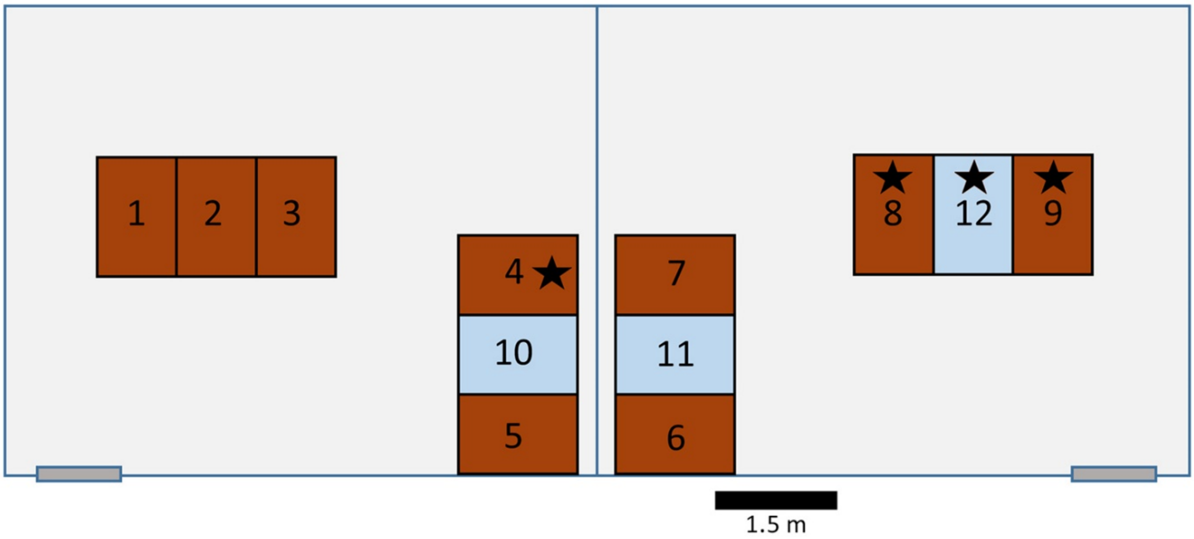

Appendix Figure 2. Arrangement of the individual cages for the raccoon dogs in 2 separate rooms of the Biosafety Level 3 facility at the Friedrich-Loeffler-Institut. Brown indicates inoculated animals, blue indicates contact animals, and stars indicate animals that were inoculated but did not show signs of infection.

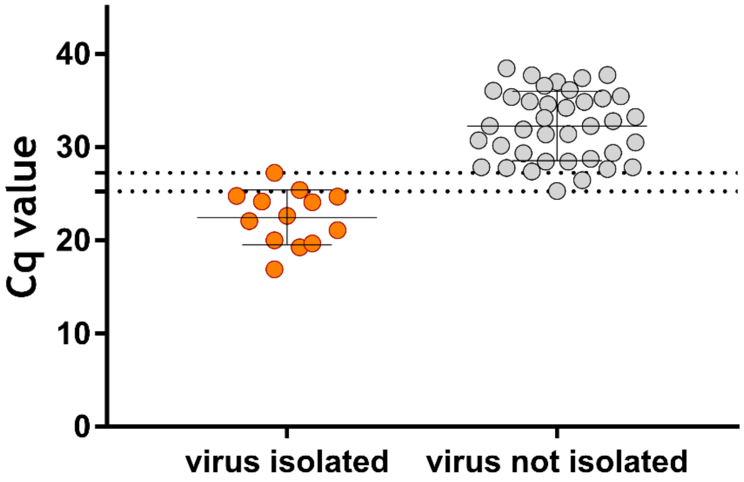

Appendix Figure 3. Cycle threshold (C_t) values of swab samples, stratified by successful virus isolation. Dashed lines indicate the upper bound of C_t values for successful virus isolation and the lower bound of C_t values for which virus isolation was not successful.

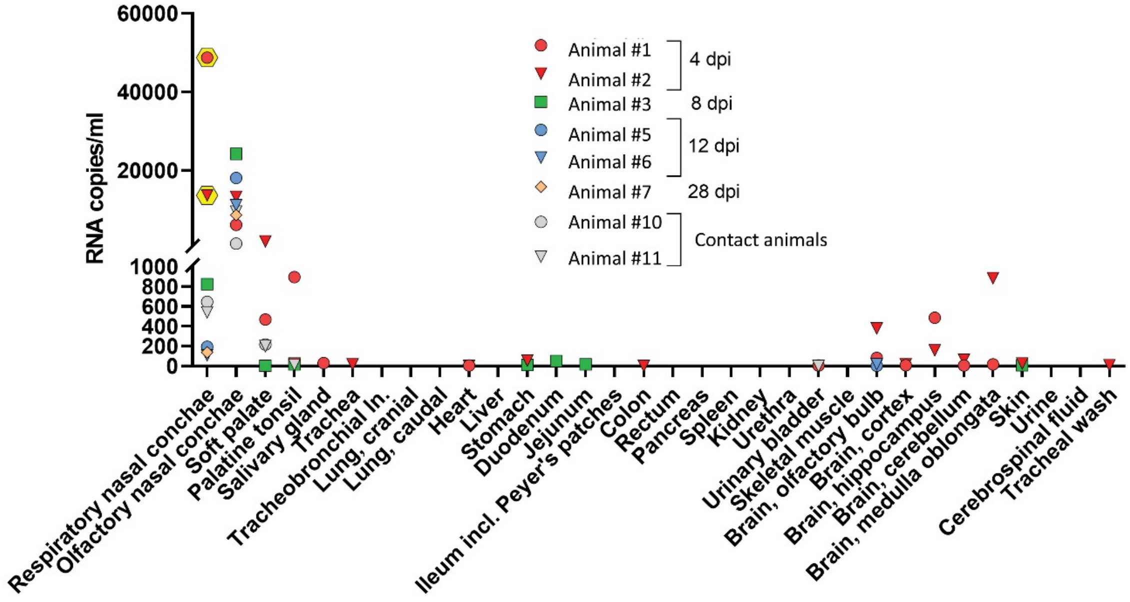

Appendix Figure 4. Viral genome loads in organs. Infectious virus was isolated only from nasal conchae on day 4 from animal nos. 1 (2.86 log_{10} 50% tissue culture infectious dose/mL) and 2 (1.63 log_{10} 50% tissue culture infectious dose/mL). Yellow hexagons indicate tissues from which virus could be isolated. 1, 0; 2, 200; 3, 400; 4, 600; 5, 600; 6, 10,000; 7, 20,000; 8, 40,000; 9, 60,000.

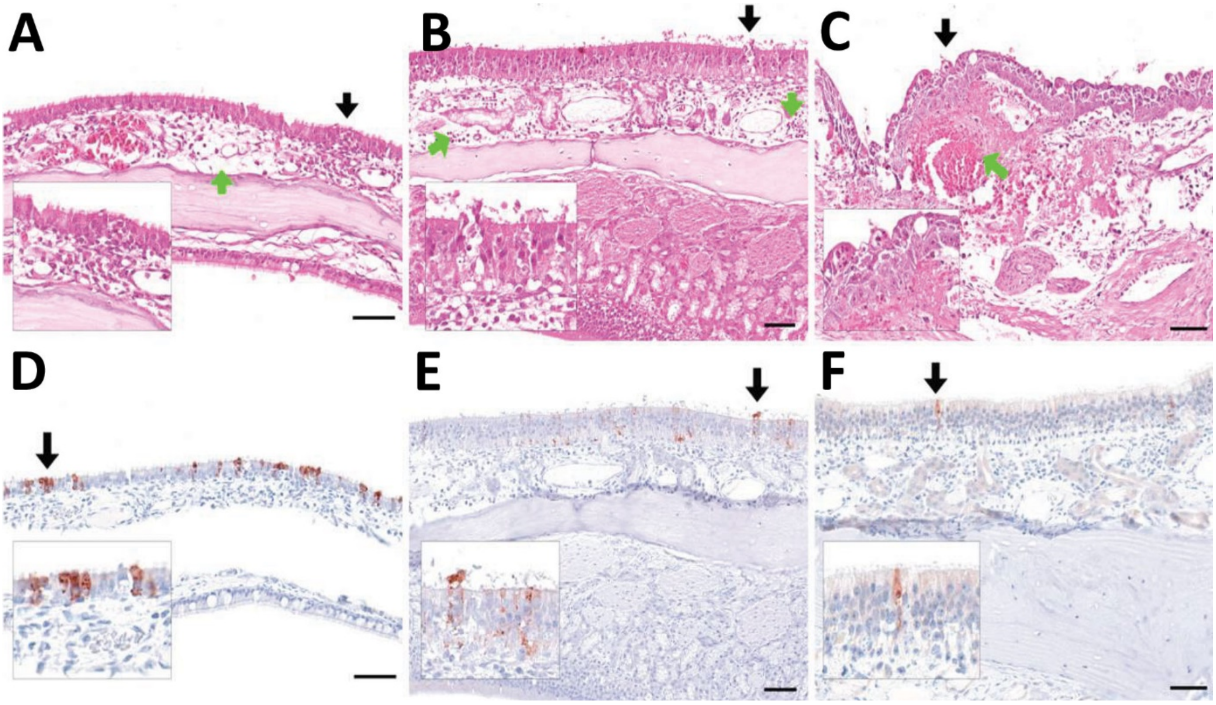

Appendix Figure 5. Lesions and antigen detection in the nasal mucosa of raccoon dogs 4 and 8 days after experimental SARS-CoV-2 infection. (A) Rhinitis, respiratory region of raccoon dog no. 1 at 4 dpi. Green arrow indicates mucosal edema; black arrow and inlay indicate epithelial degeneration with inflammation. (B) Rhinitis, olfactory region of raccoon dog no. 2 at 4 dpi. Green arrow indicates mucosal edema and inflammation; black arrow and inlay indicate epithelial necrosis and loss with minimal intraluminal debris. (C) Rhinitis, olfactory region of raccoon dog no. 3 at 8 dpi. Green arrow

indicates focal coagulative necrosis and hemorrhage; black arrow and inlay indicate epithelial necrosis with early re-epithelisation. (D) Intralesional oligofocal viral antigen in the respiratory epithelium of raccoon dog no.1 at 4 dpi, (E) Intralesional oligofocal antigen in the olfactory epithelium of raccoon dog no. 2 at 4 dpi. (F) Single antigen-positive olfactory cells of raccoon dog no. 3 at 8 dpi. A-C), hematoxylin, and eosin stain. D–F) immunohistochemical testing by avidin-biotin complex method. Red-brown indicates AEC (3-Amino-9-Ethylcarbazole) chromogen. Blue indicates Mayer's hematoxylin counter stain. All scale bars indicate 50 µm.

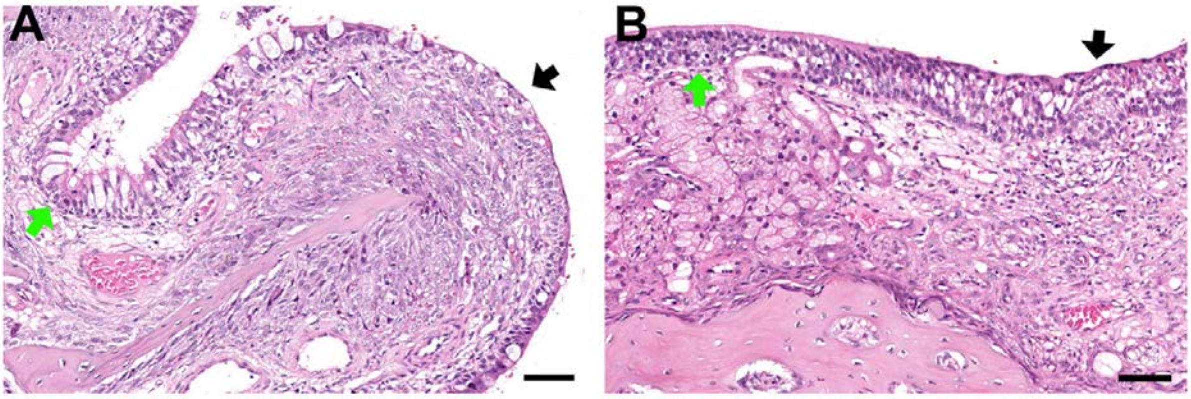

Appendix Figure 6. Lesions in the nasal mucosa of raccoon dogs 28 days after experimental SARS-CoV-2 infection. (A) Nasal mucosa, respiratory region of raccoon dog no. 7. Green arrow indicates unaffected respiratory epithelium; black arrow indicates squamous metaplasia and granulation tissue formation. (B) Nasal mucosa, olfactory region of raccoon dog no. 7. Green arrow indicates unaffected olfactory epithelium and subepithelial Bowman's glands; black arrow indicates affected irregular olfactory epithelium with loss of Bowman's glands and granulation tissue formation. Tissues stained with hematoxylin and eosin. Bar indicates 50 µm.

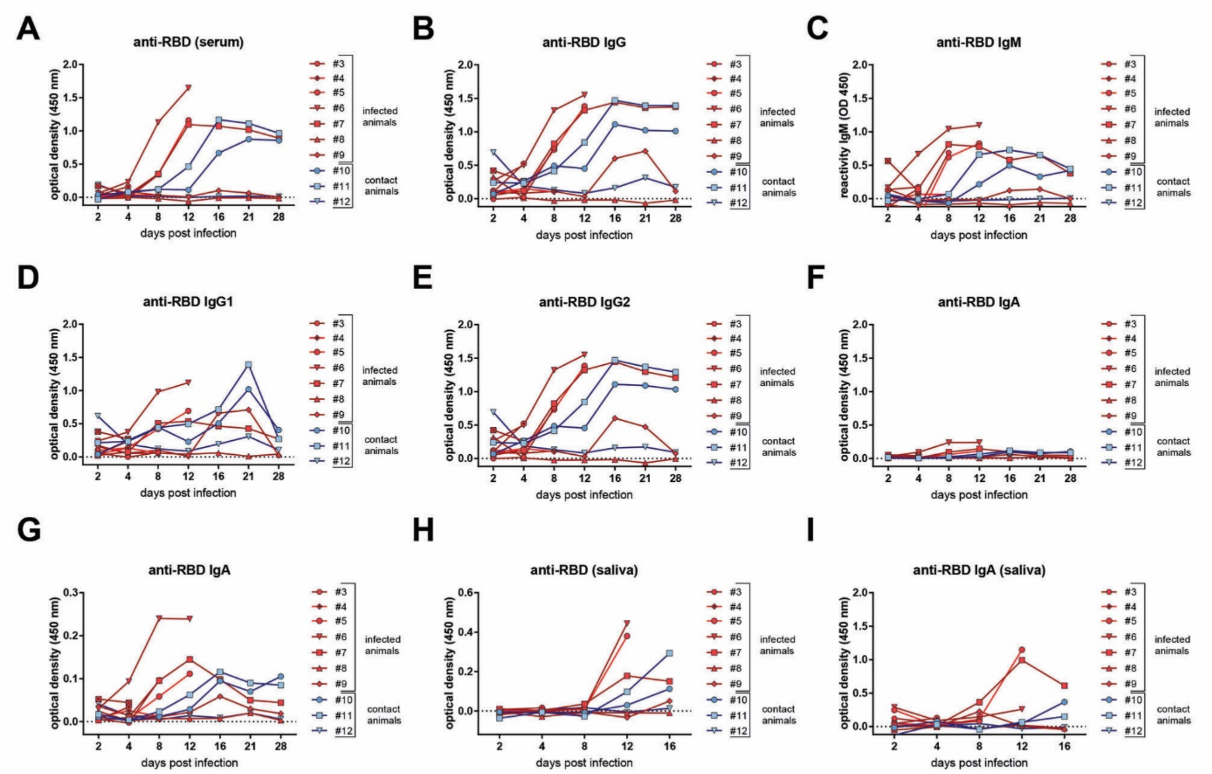

Appendix Figure 7. Antibodies against the receptor-binding domain (RBD) of SARS-Co-V-2, measured by an in-house ELISA in serum samples of raccoon dogs experimentally infected with SARS-CoV-2. (A) antibodies against RBD, using a multispecies conjugate (SBVMILK, IDvet, https://www.id-vet.com/); (B) with a conjugate against dog IgG; (C) with a conjugate against dog IgM; (D) with a conjugate against dog IgG1; (E) with a conjugate against dog IgG2; (F) with a conjugate against dog IgA, at the same scale, and (G) at a smaller scale. (H) Total antibodies against RBD in saliva; and (I) IgA against RBD in saliva.
